# Supplementary material for: Conservation laws, radiative decay rates, and excited state localization in organometallic complexes with strong spin-orbit coupling
Source: Sci Rep. 2015 Jun 30;5:10815. doi: 10.1038/srep10815 (PMC4485171; doi:10.1038/srep10815)
Supplement: Supplementary Information [file srep10815-s1.pdf]

Supplementary information

Conservation laws, radiative decay rates, and excited state localization in organometallic complexes with strong spin-orbit coupling

Benjamin J. Powell

Centre for Organic Photonics and Electronics, School of Mathematics and Physics, The University of Queensland, QLD 4072, Australia

bjpowell@gmail.com

Results for other parameters

Figs. S1-S4 show the $\mathfrak{L}^z = 1$ solutions of the pseudo-angular momentum model of a trigonal complex, $H = H_o + H_t$, for wide ranges of parameters.

Figs. S5-S9 show all $\mathfrak{L}^z = 1$ solutions of the full model, $H = H_o + H_t + H_{JT}$, for wide ranges of parameters. Figs. S10-S14 show the same data but focus only on the substates of T_1, i.e., the three lowest energy solutions. In Figs S15-19 we plot the radiative rates for the substates of T_1 given the same parameters using the approximations described in the main text.

It can be seen from these figures that the main results – particularly the selection rules (conservation laws), the pattern of zero-field splitting of T_1 ($E_{I,II} < E_{II,III}$) and the relative radiative decay rates ($k_R^I < k_R^{II} < k_R^{III}$) hold whatever parameter values are relevant. This is consistent with these trends being observed experimentally in the wide range of complexes detailed in Table 1 of the main text.

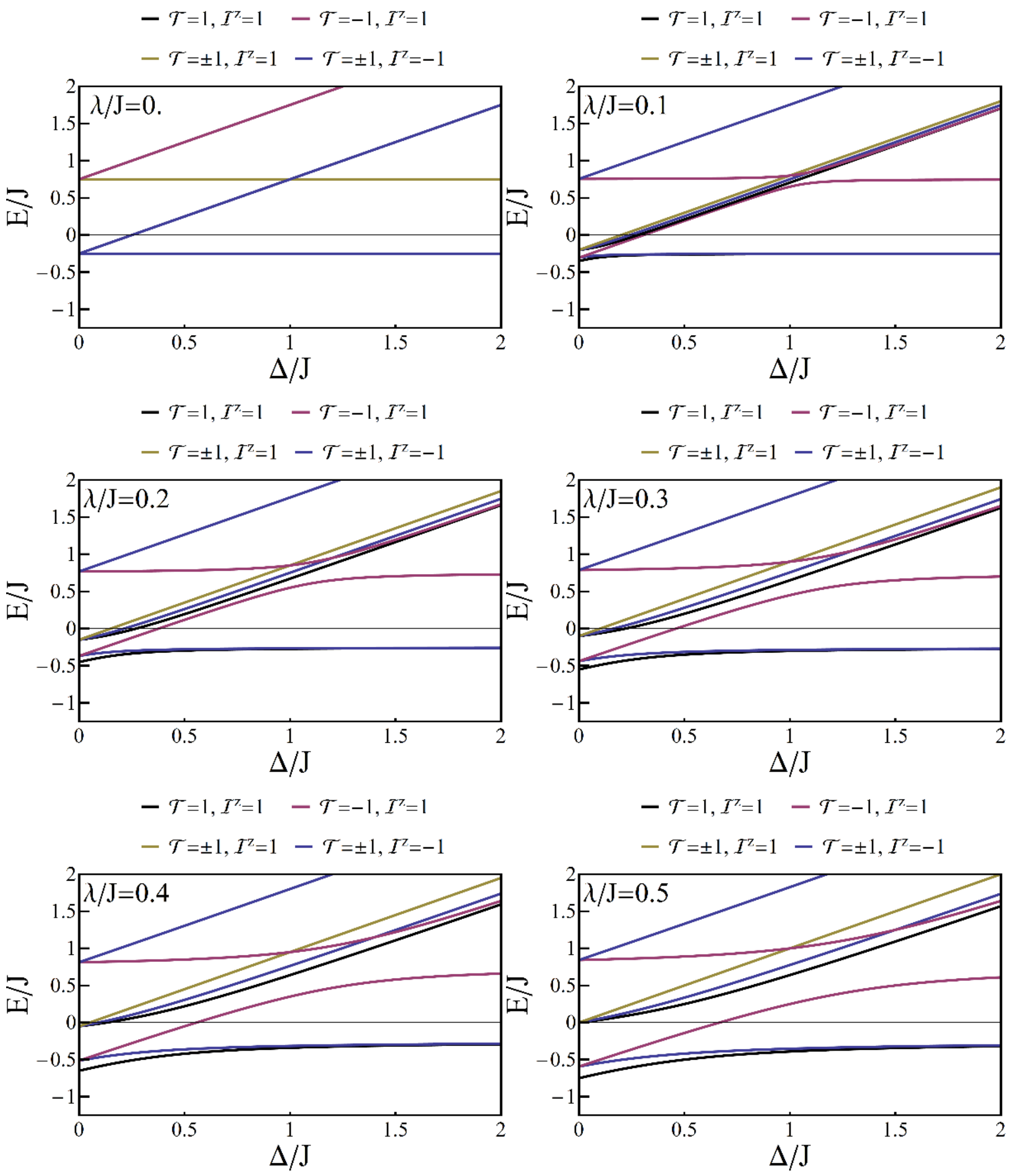

Fig. S1. Solution of the pseudo-angular momentum model of a trigonal complex, $H = H_o + H_t$, spectra for selected λ varying Δ/J. For $\lambda = 0$ some lines are obscured by others.

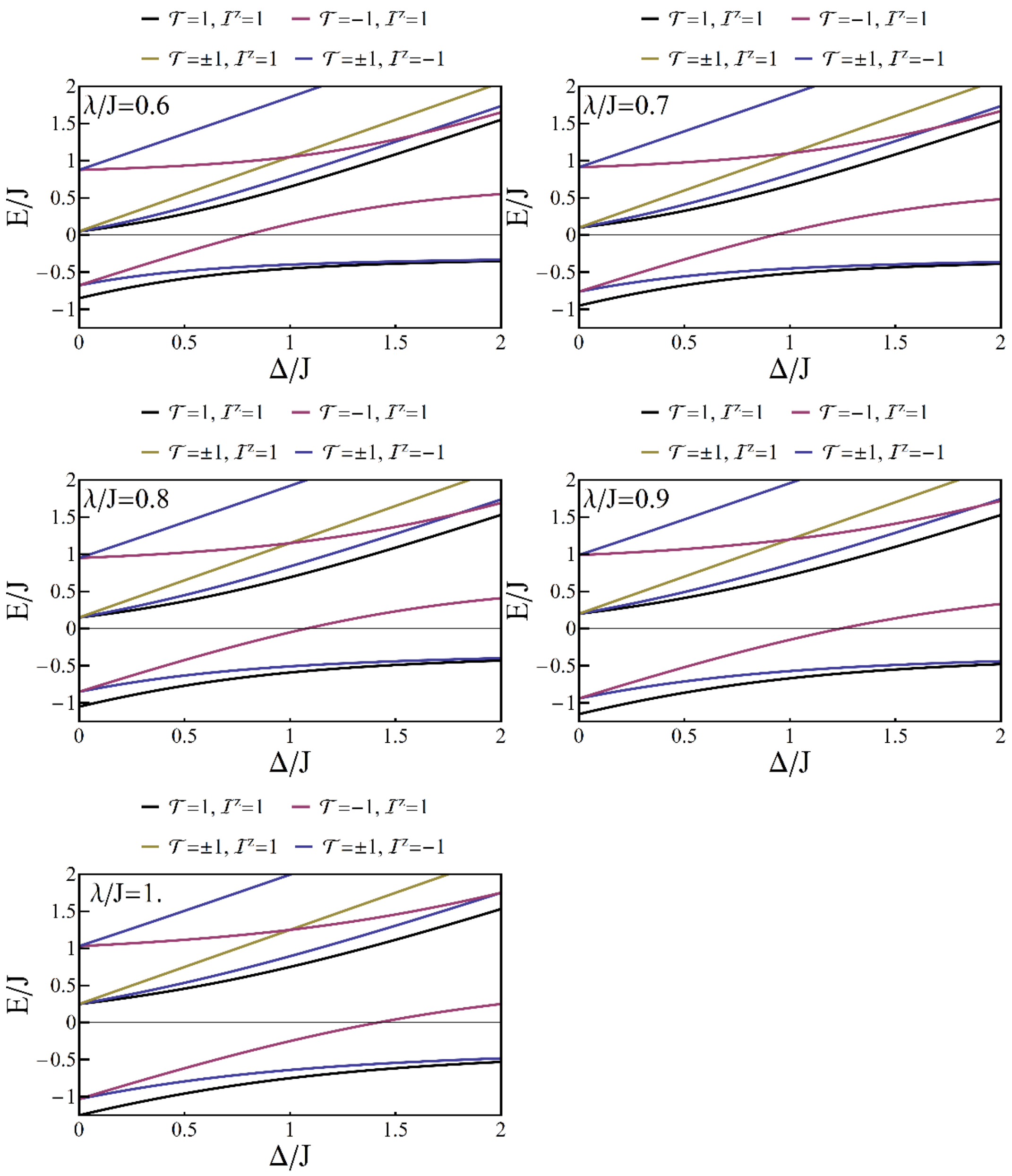

Fig. S2. Solution of the pseudo-angular momentum model of a trigonal complex, $H = H_o + H_t$, spectra for selected λ varying Δ/J.

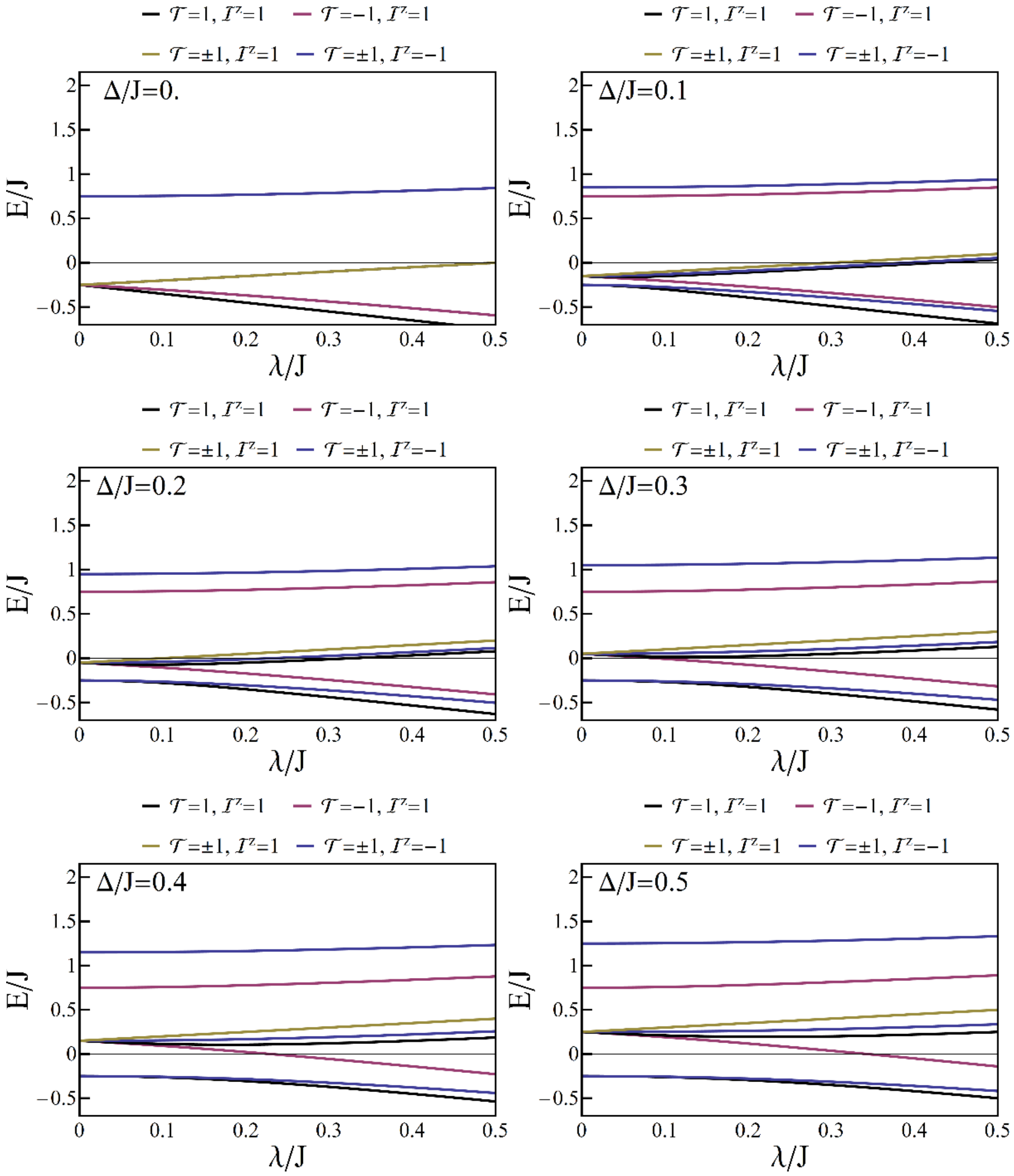

Fig. S3. Solution of the pseudo-angular momentum model of a trigonal complex, $H = H_o + H_t$, spectra for selected Δ varying λ/J. For $\Delta = 0$ some lines are obscured by others.

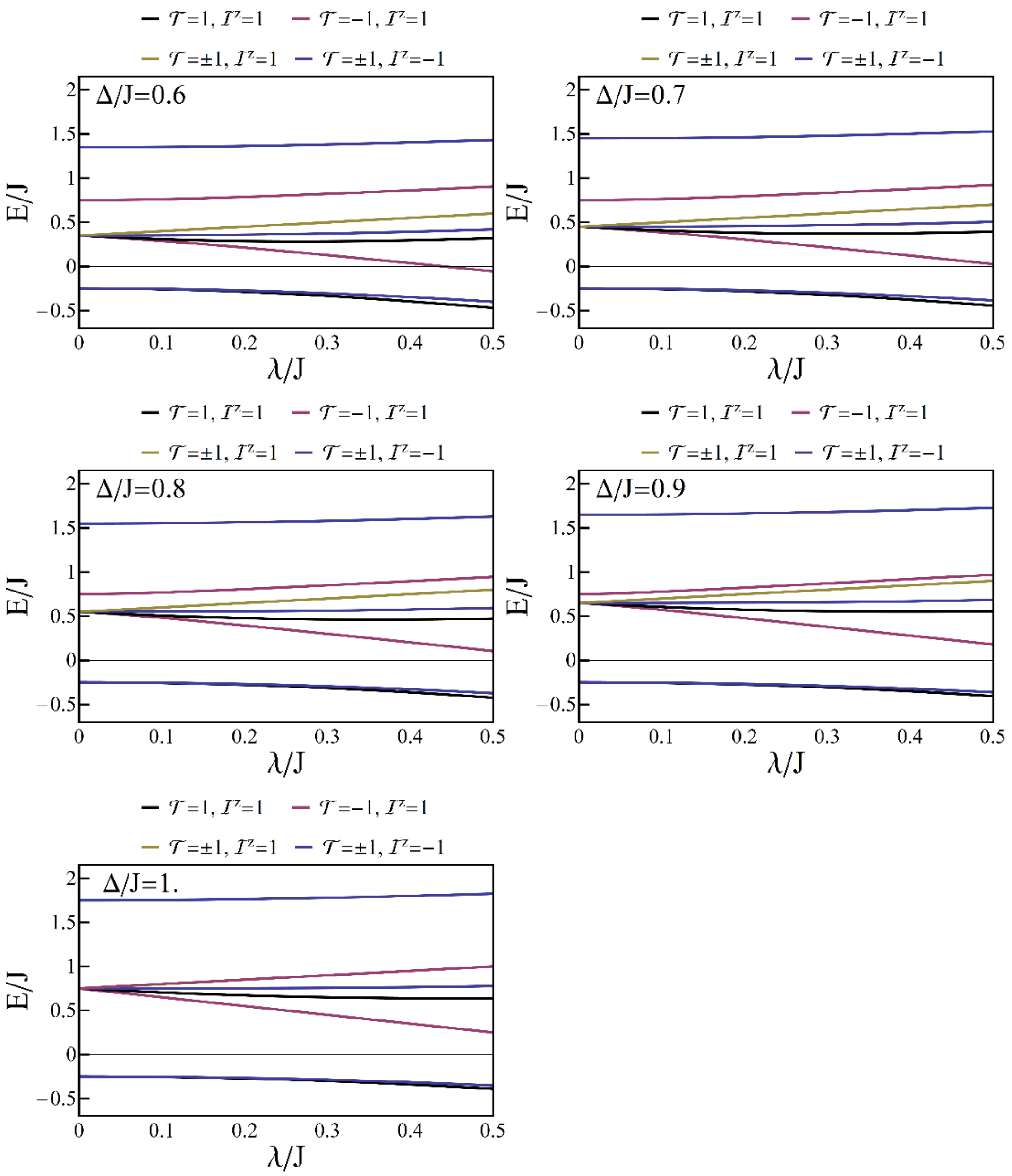

Fig. S4. Solution of the pseudo-angular momentum model of a trigonal complex, $H = H_o + H_t$, spectra for selected Δ varying λ/J. For $\Delta = J$ some lines are obscured by others.

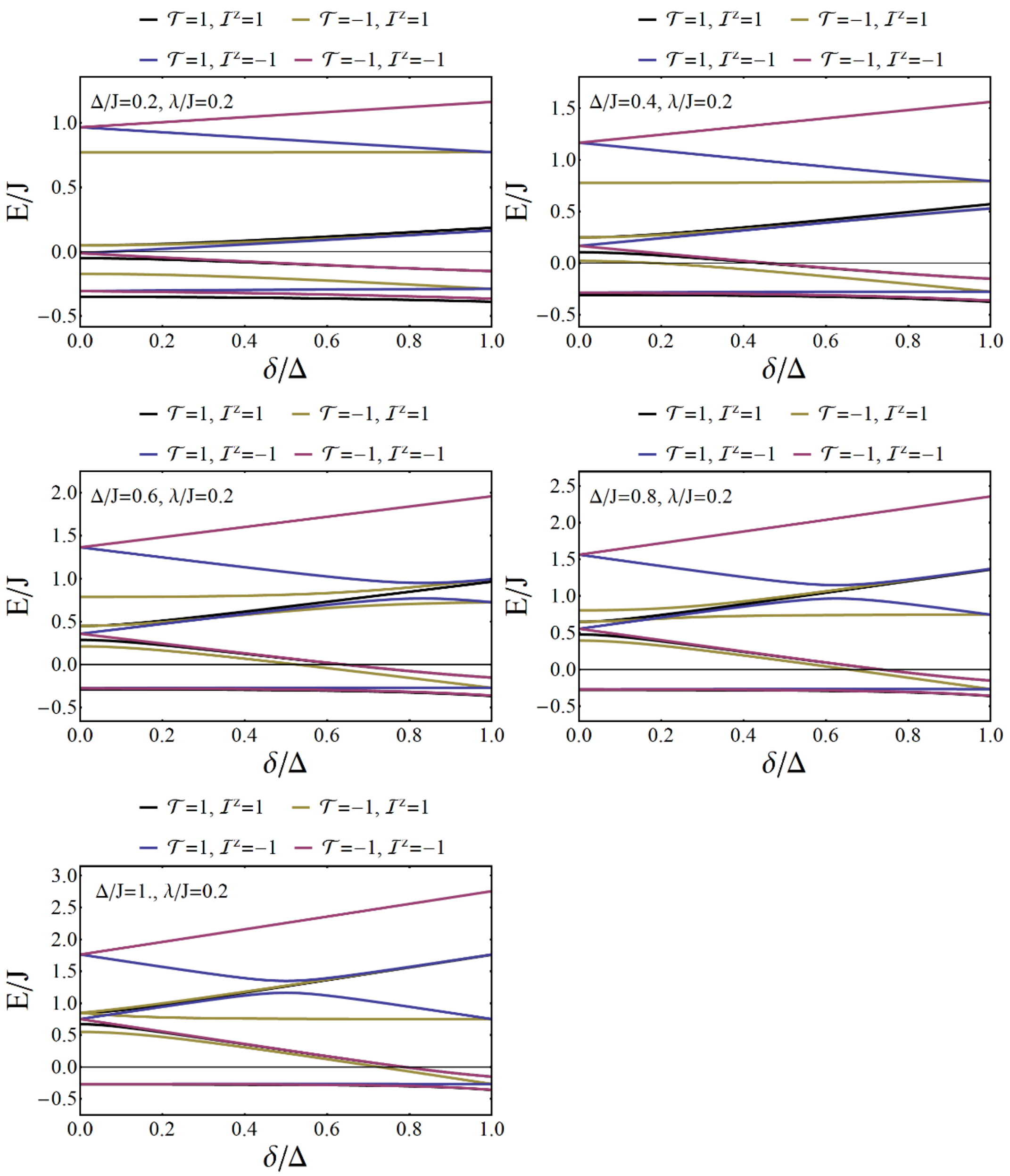

Fig. S5. Solution of the full model, $H = H_o + H_t + H_{JT}$, for selected parameters. Only the $\mathfrak{L}^z = 1$ solutions are shown.

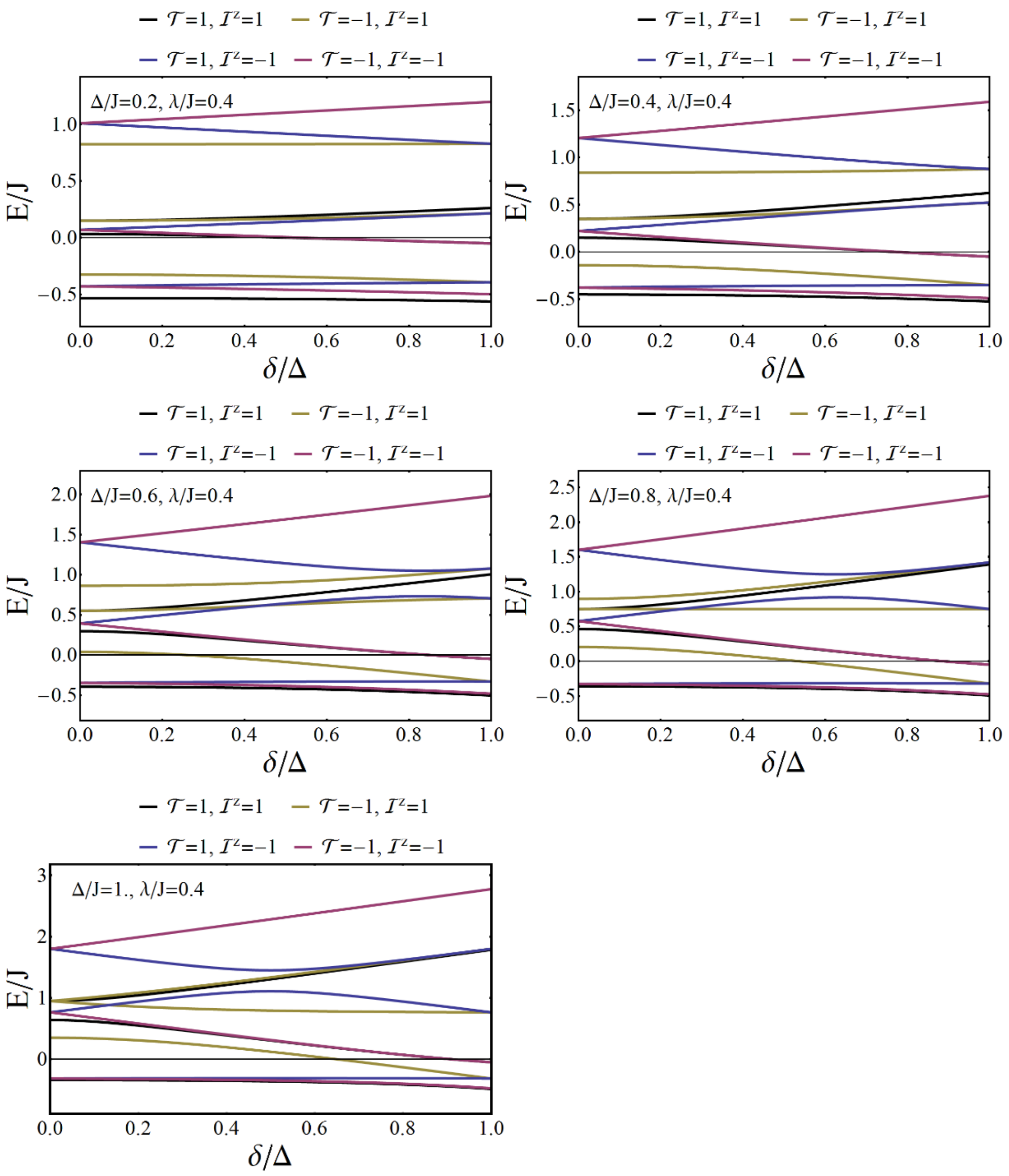

Fig. S6. Solution of the full model, $H = H_o + H_t + H_{JT}$, for selected parameters. Only the $\mathfrak{L}^z = 1$ solutions are shown.

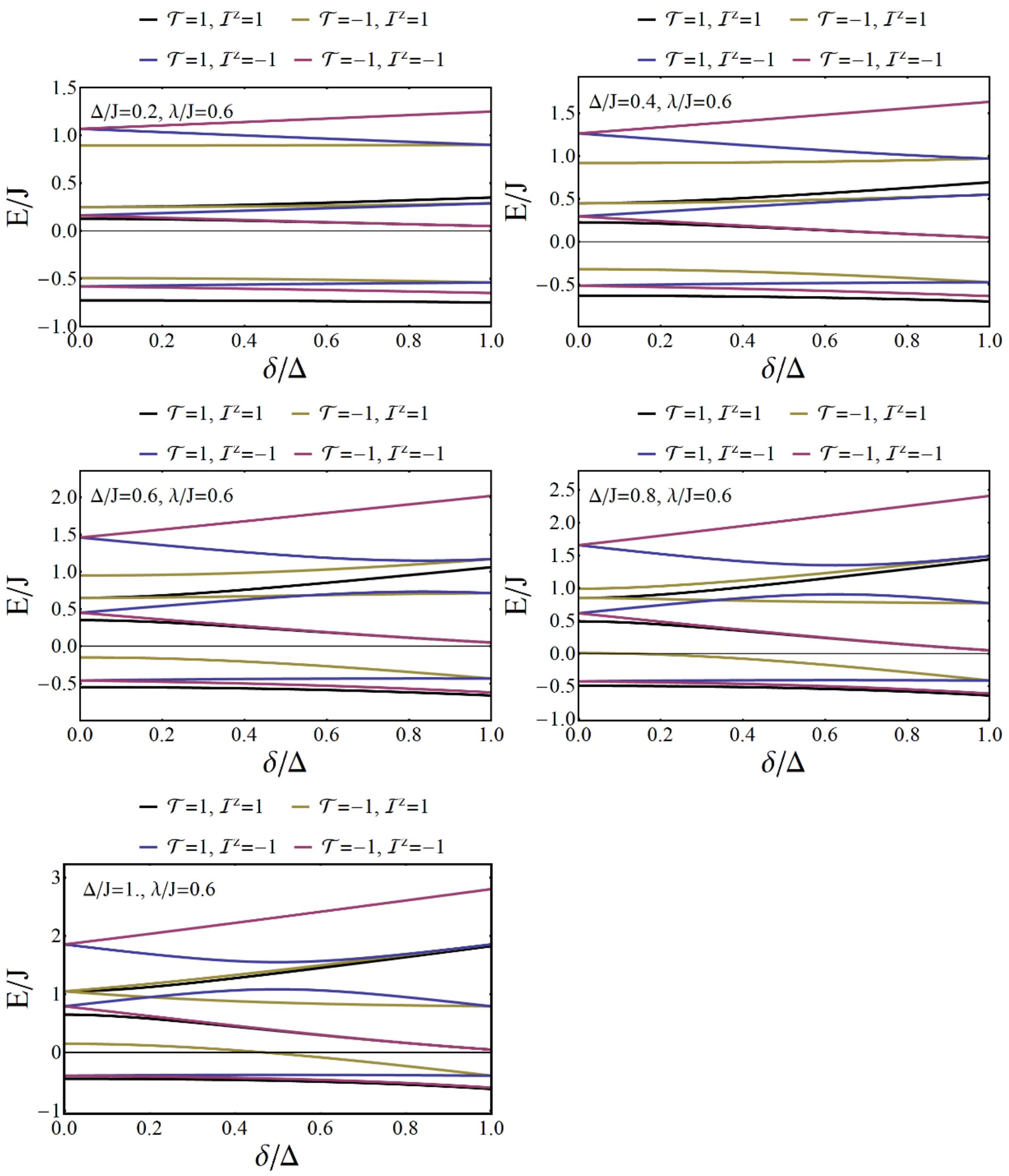

Fig. S7. Solution of the full model, $H = H_o + H_t + H_{JT}$, for selected parameters. Only the $\mathfrak{L}^z = 1$ solutions are shown.

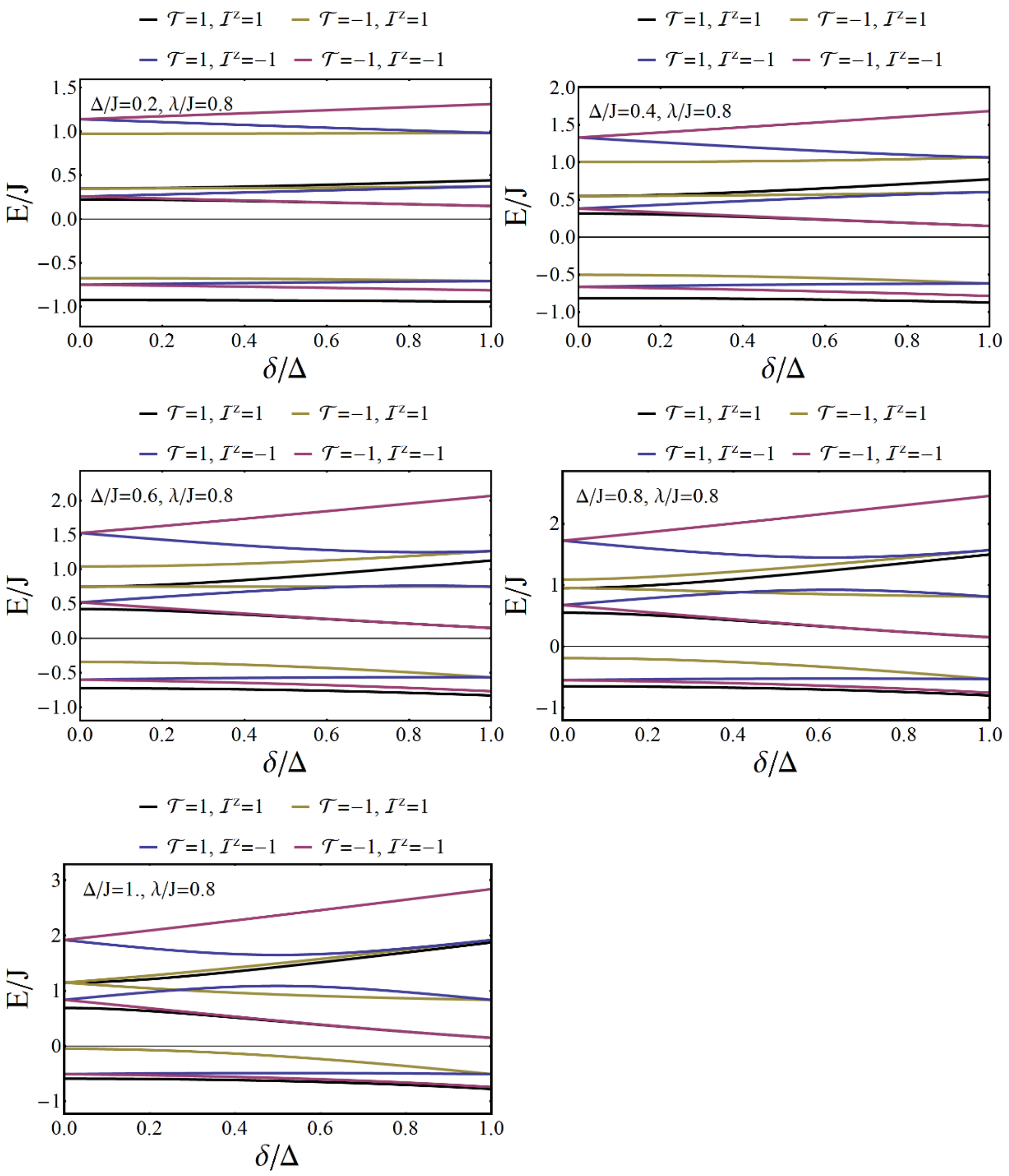

Fig. S8. Solution of the full model, $H = H_o + H_t + H_{JT}$, for selected parameters. Only the $\mathfrak{L}^z = 1$ solutions are shown.

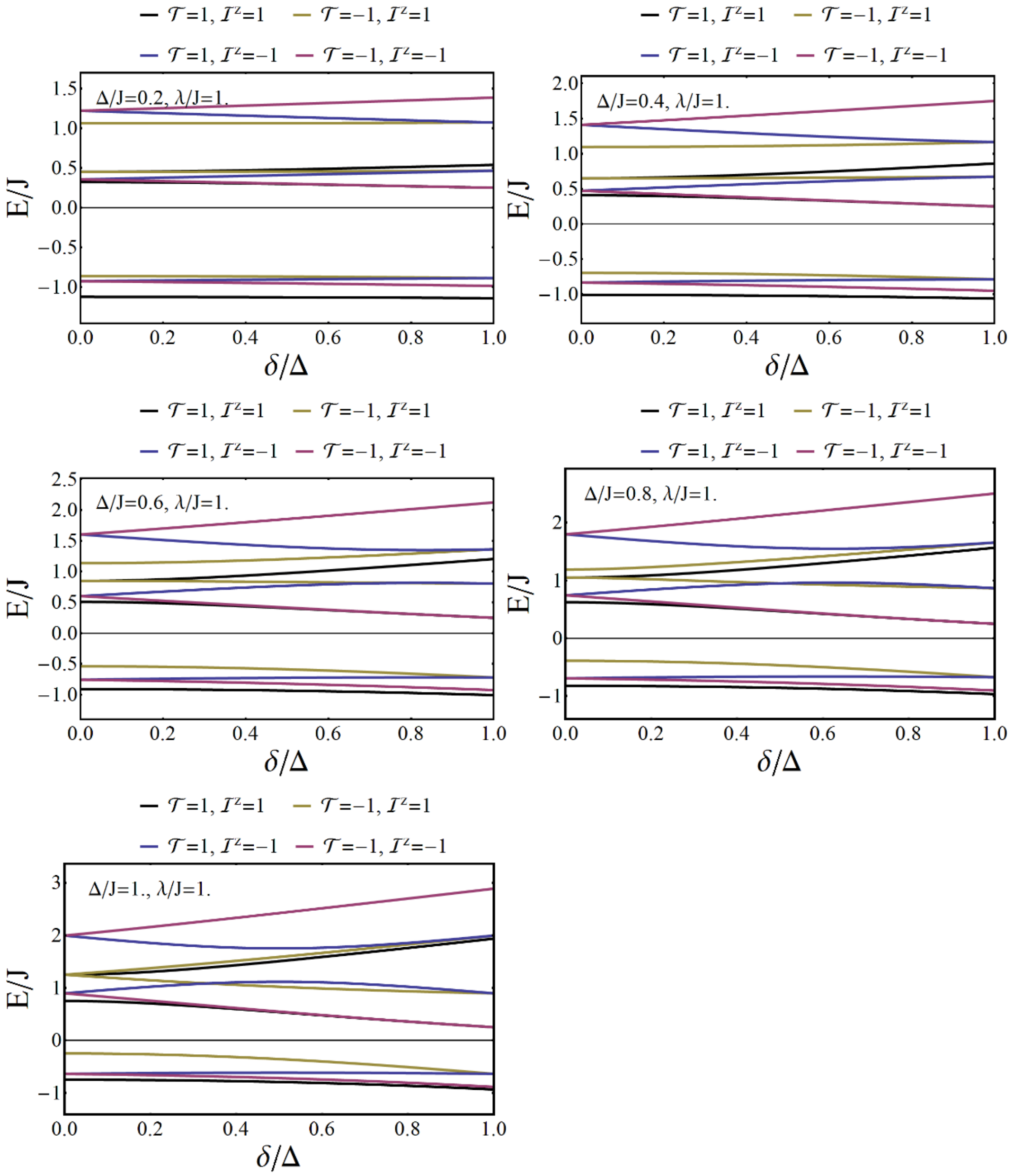

Fig. S9. Solution of the full model, $H = H_o + H_t + H_{JT}$, for selected parameters. Only the $\mathfrak{L}^z = 1$ solutions are shown.

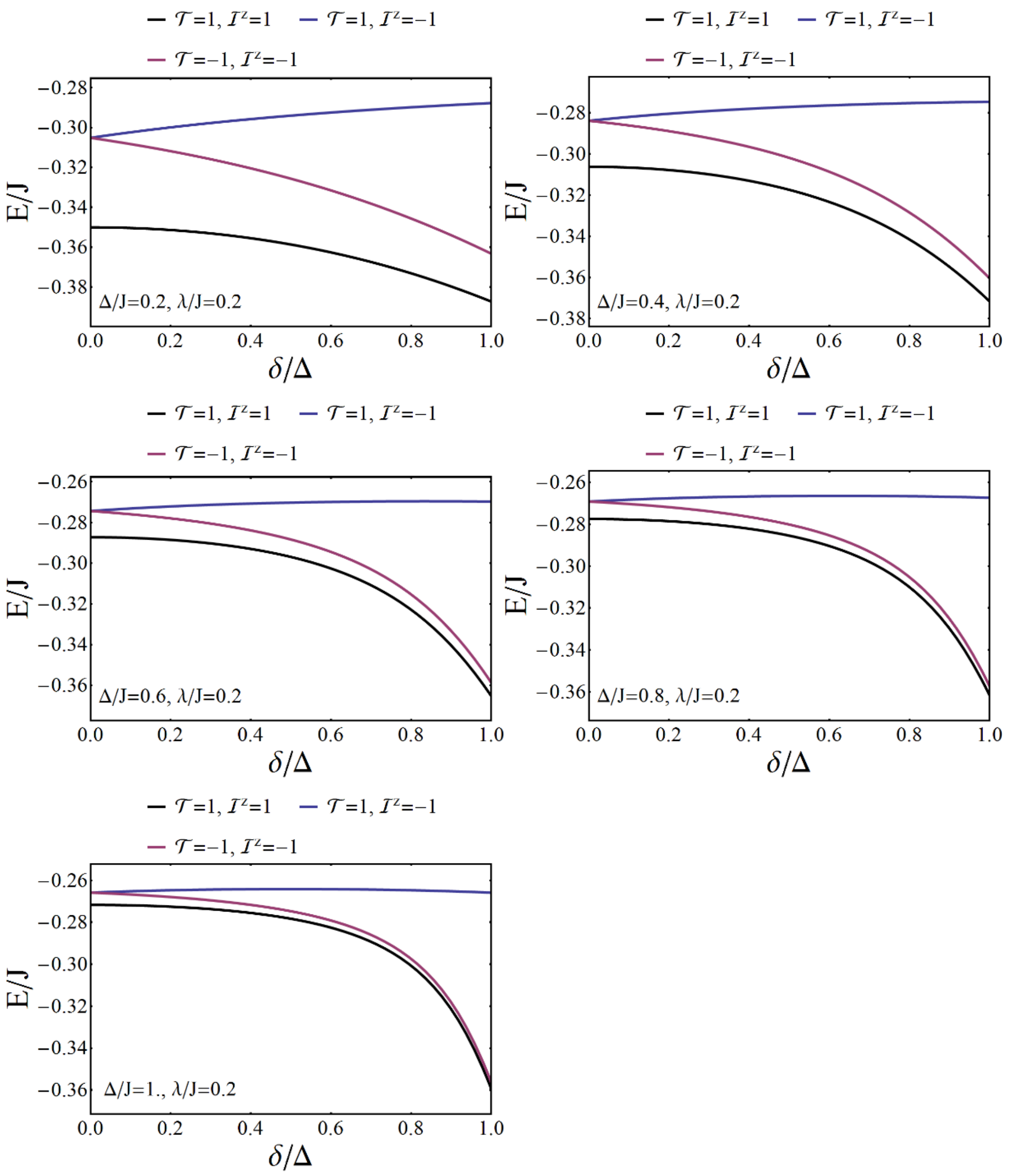

Fig. S10. The three lowest energy solutions, i.e., the substates of T_1, for the full model, $H = H_o + H_t + H_{JT}$, for the same parameters as studied in Fig. S5.

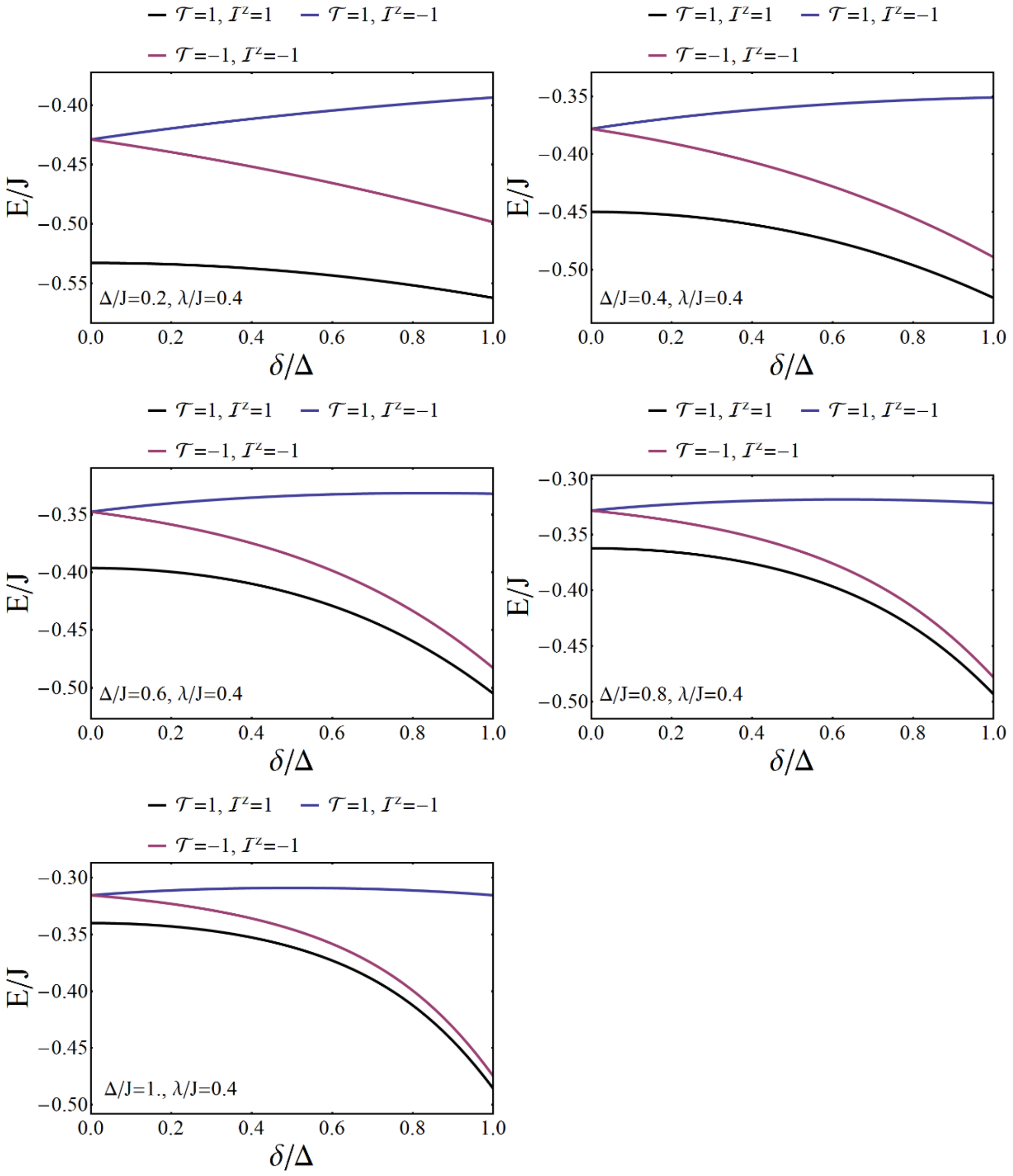

Fig. S11. The three lowest energy solutions, i.e., the substates of T_1, for the full model, $H = H_o + H_t + H_{JT}$, for the same parameters as studied in Fig. S6.

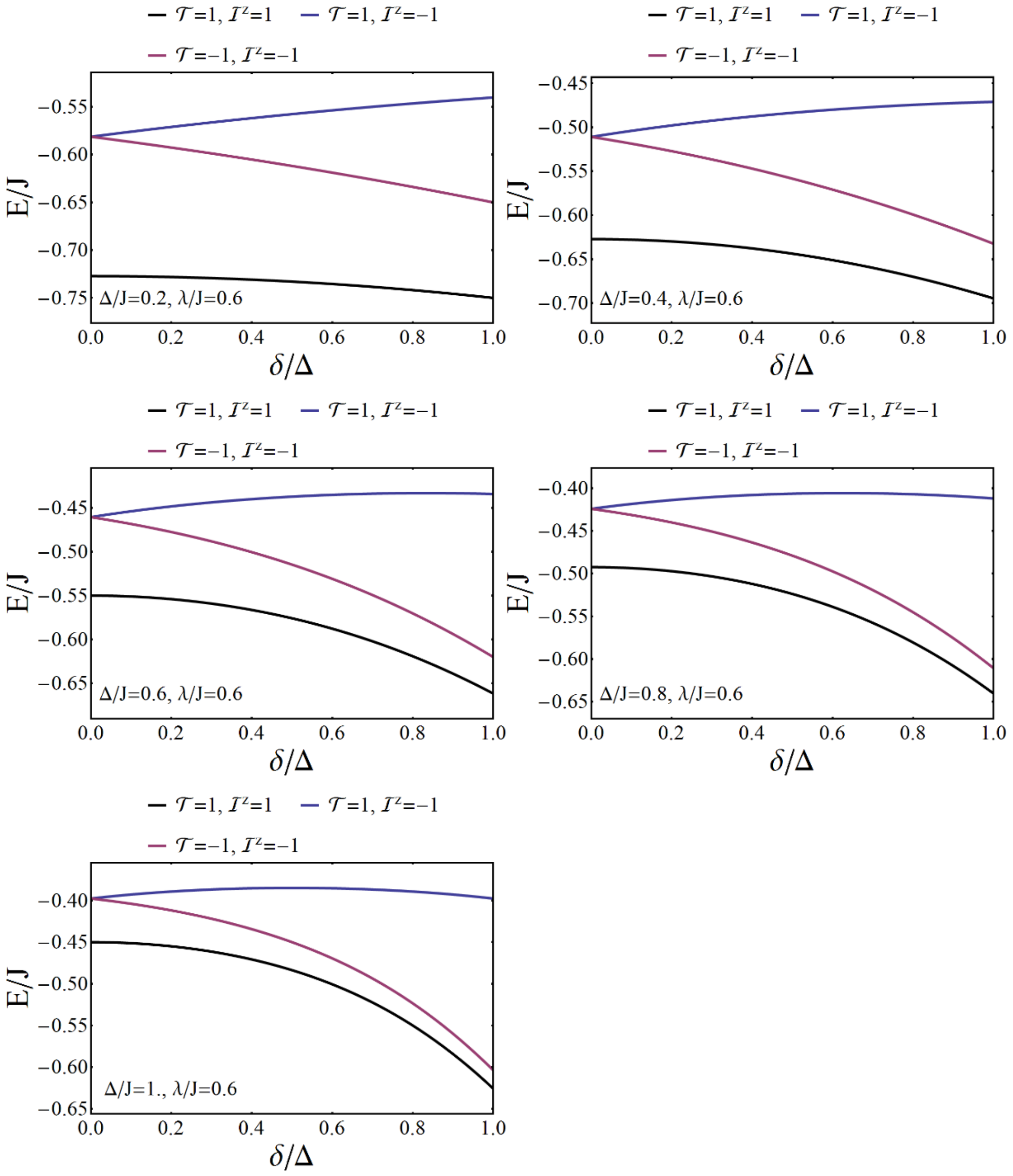

Fig. S12. The three lowest energy solutions, i.e., the substates of T_1, for the full model, $H = H_o + H_t + H_{JT}$, for the same parameters as studied in Fig. S7.

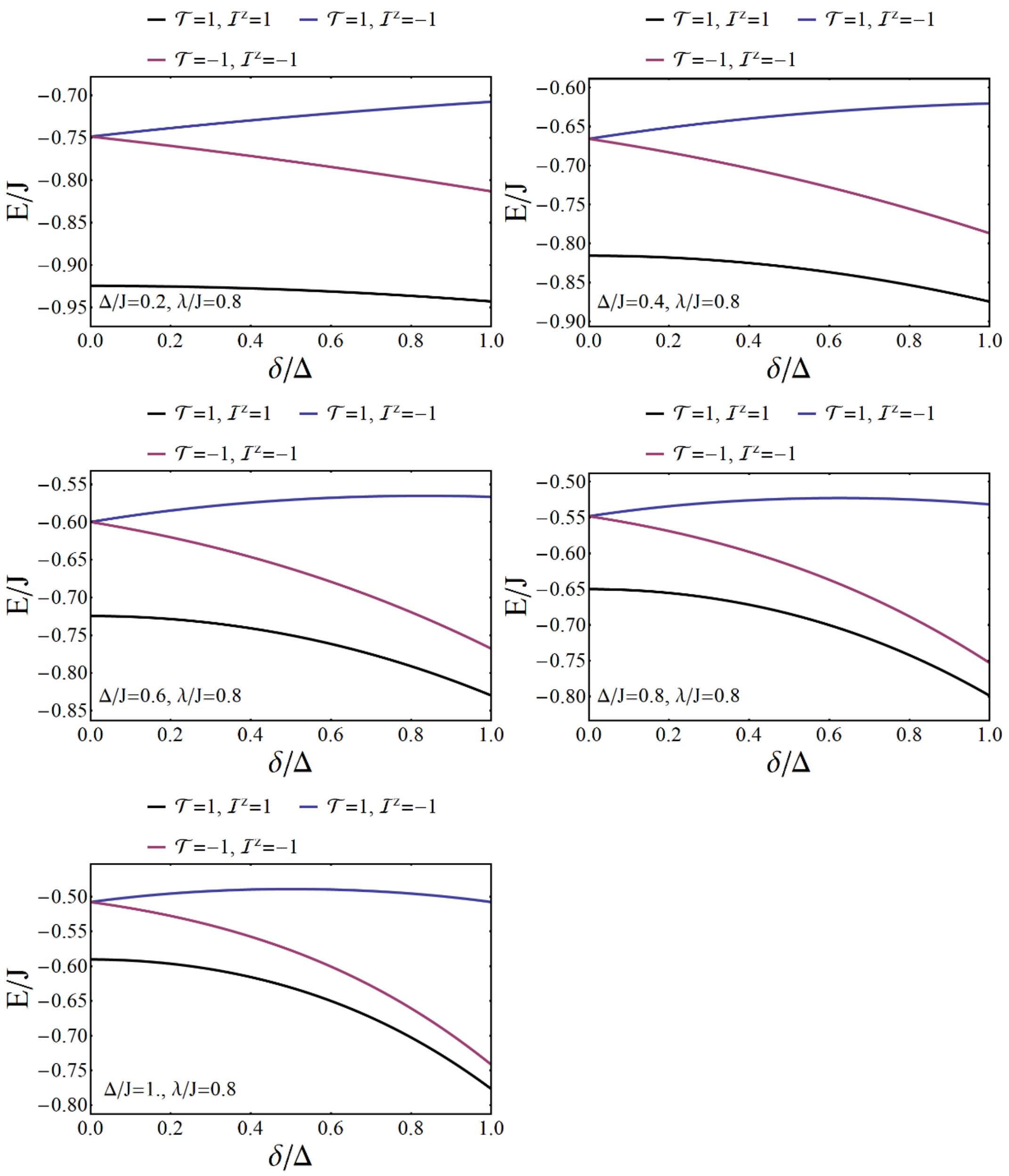

Fig. S13. The three lowest energy solutions, i.e., the substates of T_1, for the full model, $H = H_o + H_t + H_{JT}$, for the same parameters as studied in Fig. S8.

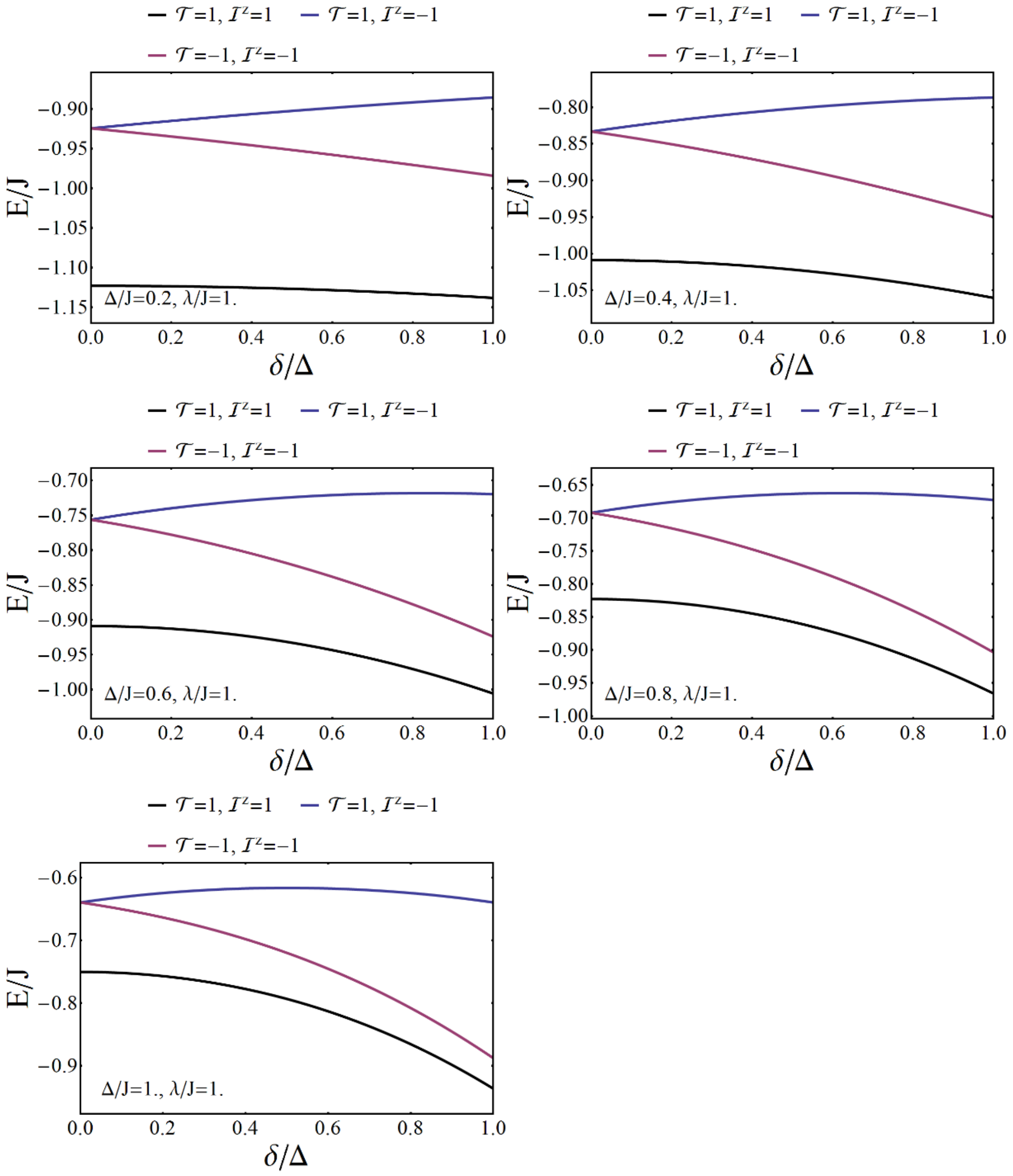

Fig. S14. The three lowest energy solutions, i.e., the substates of T_1, for the full model, $H = H_o + H_t + H_{JT}$, for the same parameters as studied in Fig. S9.

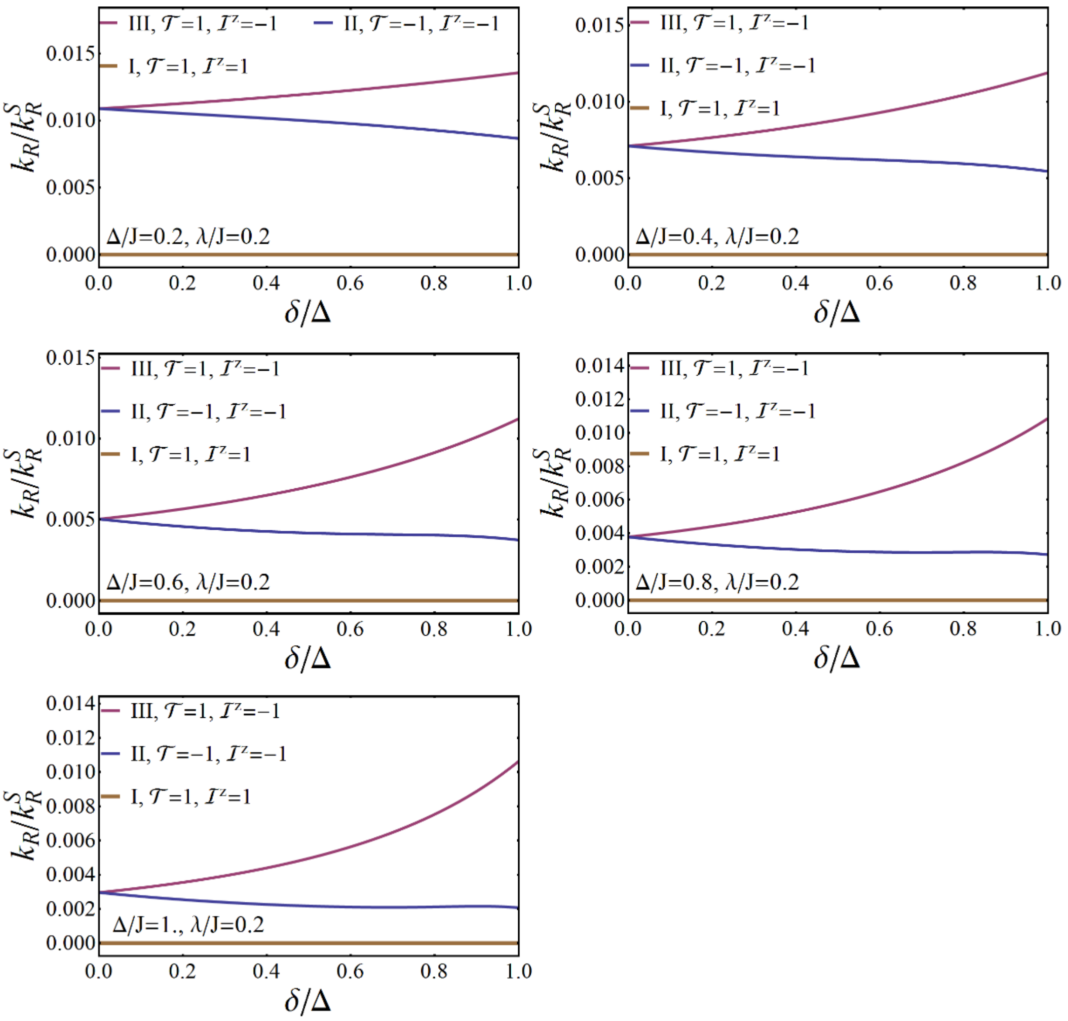

Fig. S15. Radiative rates of the three lowest energy solutions, i.e., the substates of T_1, for the full model, $H = H_o + H_t + H_{JT}$, for the same parameters as studied in Figs. S5 and S10.

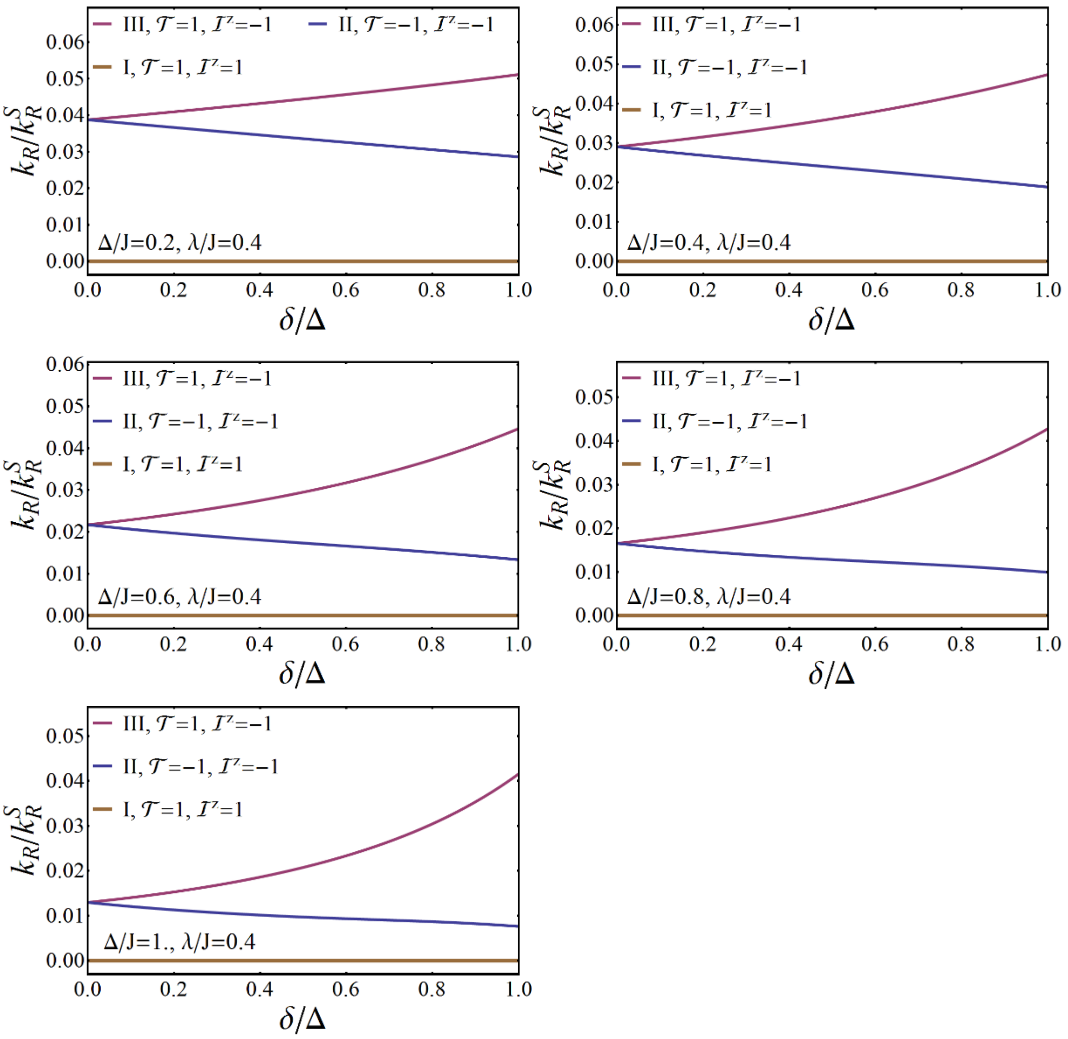

Fig. S16. Radiative rates of the three lowest energy solutions, i.e., the substates of T_1, for the full model, $H = H_o + H_t + H_{JT}$, for the same parameters as studied in Figs. S6 and S11.

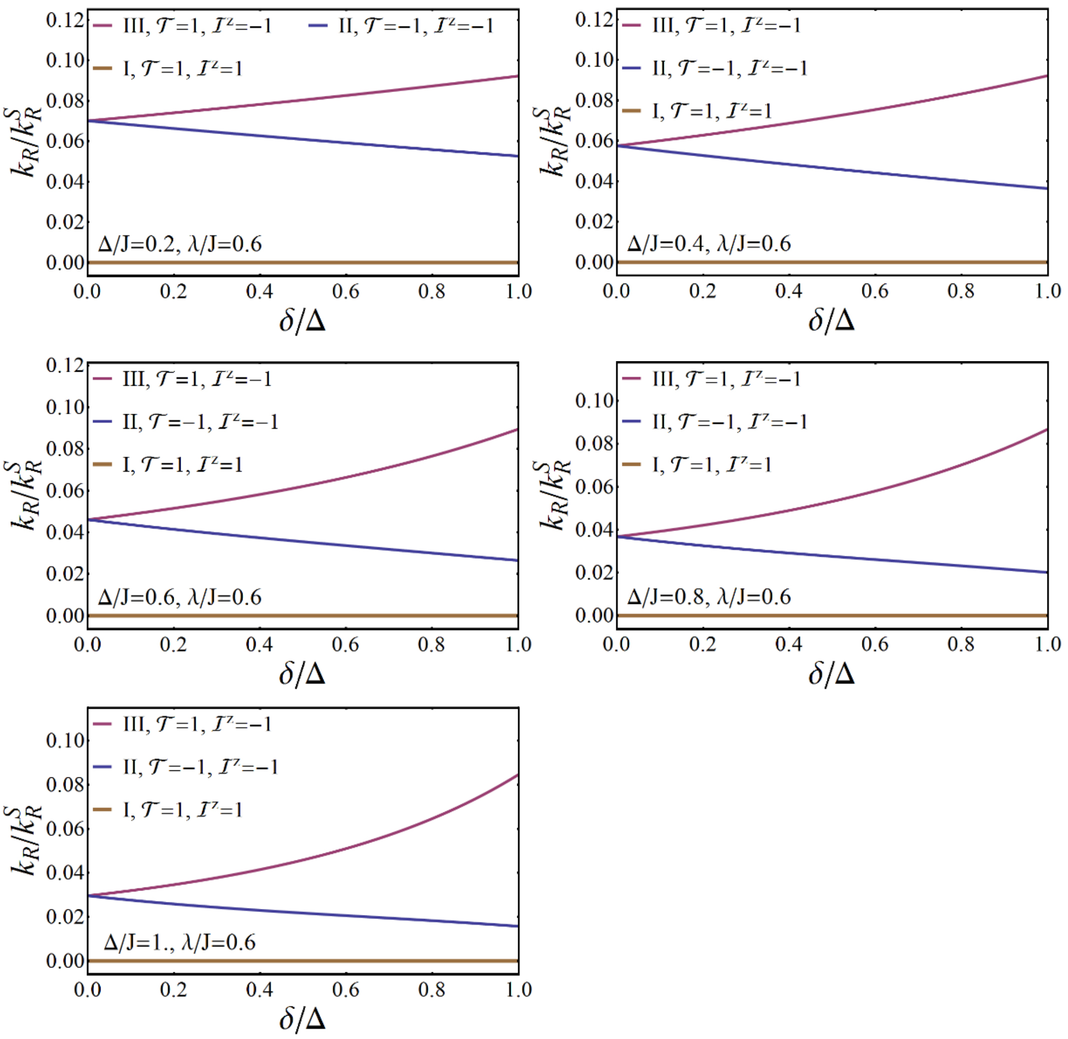

Fig. S17. Radiative rates of the three lowest energy solutions, i.e., the substates of T_1, for the full model, $H = H_o + H_t + H_{JT}$, for the same parameters as studied in Figs. S7 and S12.

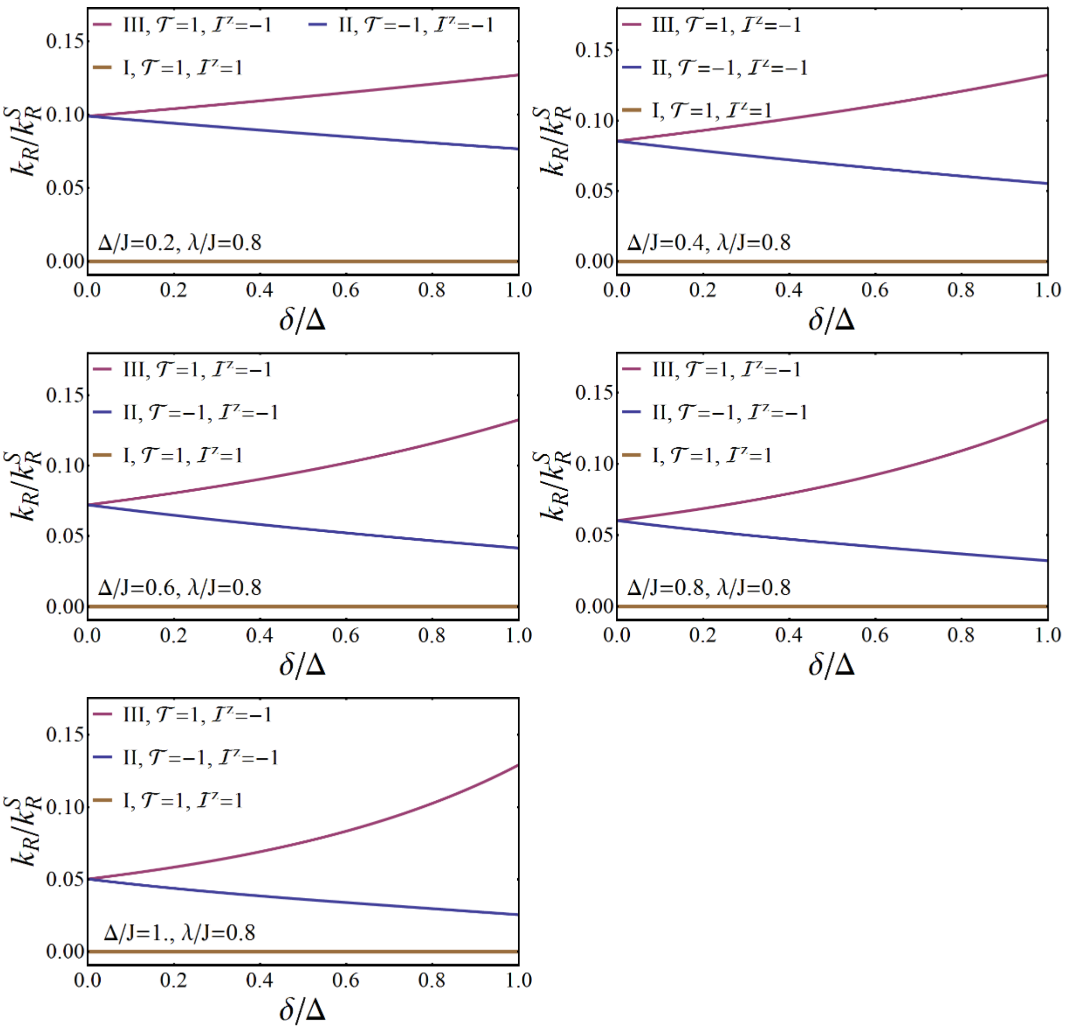

Fig. S18. Radiative rates of the three lowest energy solutions, i.e., the substates of T_1, for the full model, $H = H_o + H_t + H_{JT}$, for the same parameters as studied in Figs. S8 and S13.

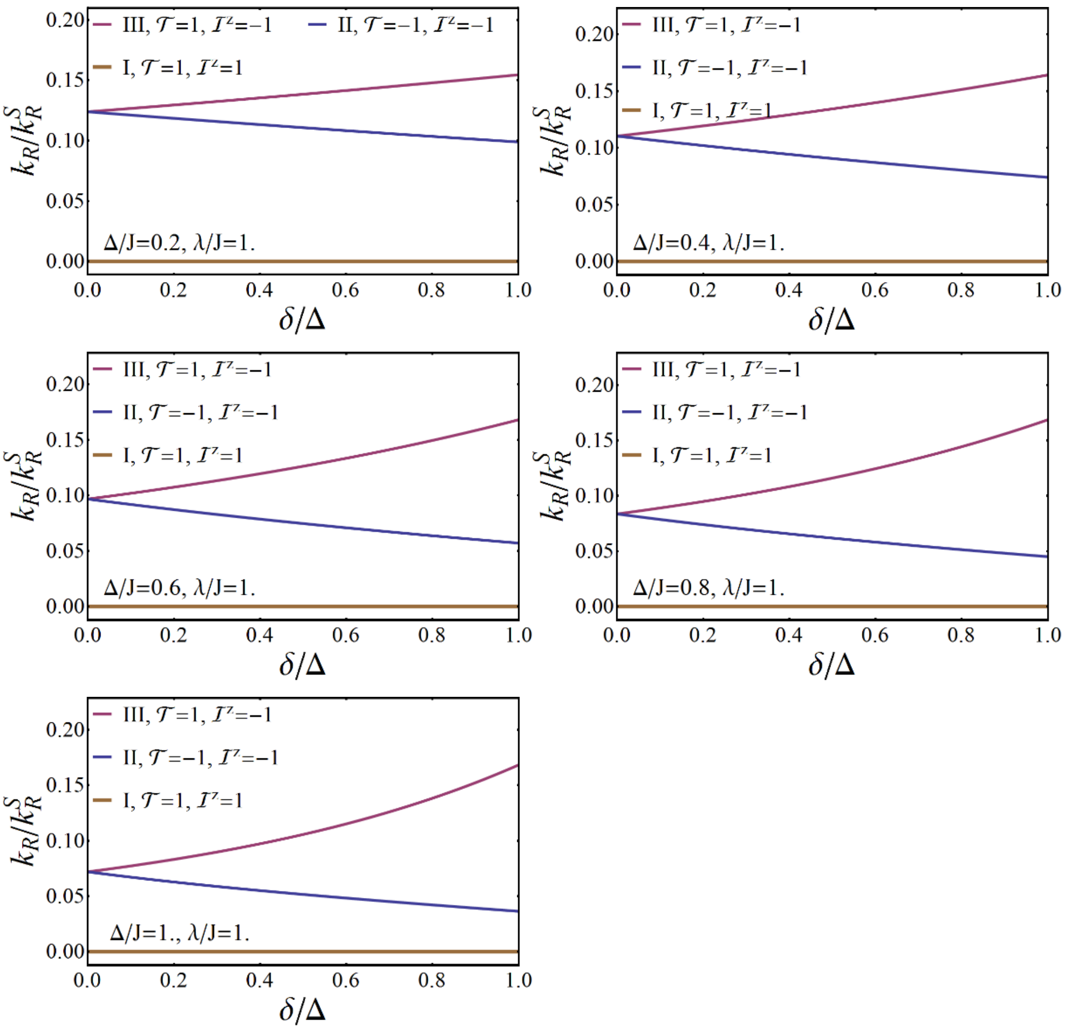

Fig. S19. Radiative rates of the three lowest energy solutions, i.e., the substates of T_1, for the full model, $H = H_o + H_t + H_{JT}$, for the same parameters as studied in Figs. S9 and S14.
